# Acute Effects of Transdermal Administration of Jojoba Oil on Lipid Metabolism in Mice

**DOI:** 10.3390/medicina55090594

**Published:** 2019-09-15

**Authors:** Yutaka Matsumoto, Sihui Ma, Takaki Tominaga, Keiko Yokoyama, Kanae Kitatani, Kazumasa Horikawa, Katsuhiko Suzuki

**Affiliations:** 1 Faculty of Nursing, Tokai University School of Medicine, Isehara, Kanagawa 259-1292, Japan; matu@tsc.u-tokai.ac.jp (Y.M.); horikawa_kaz@hotmail.com (K.H.); 2 Graduate School of Sports Sciences, Waseda University, Tokorozawa, Saitama 359-1192, Japan; masihui@toki.waseda.jp (S.M.); takaki.k-bbc@akane.waseda.jp (T.T.); 3 Support Center for Medical Research and Education, Tokai University, Isehara, Kanagawa 259-1292, Japan; kekoyoko@tsc.u-tokai.ac.jp (K.Y.); kitakana@tsc.u-tokai.ac.jp (K.K.); 4 Faculty of Sport Sciences, Waseda University, Tokorozawa, Saitama 359-1192, Japan

**Keywords:** jojoba oil, fatty acid, non-esterified fatty acids (NEFA), aromatherapy, lipid metabolism, transdermal absorption

## Abstract

*Background and objectives:* Aroma therapy is a complementary therapy using essential oils diluted with carrier oils. Jojoba oils have been widely used as carrier oils. However, limited information is available regarding their effects on blood biochemical parameters. This study aimed to investigate the effect of transdermal administration of jojoba oil on blood biochemical parameters in mice. *Materials and Methods:* Eight-week-old male hairless mice were randomly divided into naïve control and treatment groups. In the treatment group, mice were topically administered 4 μL of jojoba oil, per gram of body weight, on the dorsa 30 min before euthanasia. Thereafter, serum biochemical parameters were assayed, and gene expression was analyzed in various tissues via a real-time polymerase chain reaction. *Results:* Serum non-esterified fatty acid (NEFA) levels increased significantly 30 min after topical application of jojoba oil (*p* < 0.05). *Atgl* was significantly upregulated in the liver (*p* < 0.05), and *Atgl* upregulation in the liver was positively correlated with serum NEFA levels (*r* = 0.592, *p* < 0.05). Furthermore, a trend of decreasing fatty acid trafficking-related gene (*FABPpm*, *FATP-1*, *FATP-3*, and *FATP-4*) expression in the skin after topical application of jojoba oil (*p* = 0.067, 0.074, 0.076, and 0.082, respectively) was observed. *Conclusions:* Serum NEFA levels were elevated 30 min after transdermal administration of jojoba oil. The mechanisms of elevated serum NEFA levels might be related to both enhanced lipolysis in the liver and reduced fatty acid trafficking in the skin.

## 1. Introduction

Plant-based therapies for many diseases have been studied for a long time. Research is currently being conducted to assess the application of plant-derived components such as extracts and essential oils for medical treatments [1,2] and insecticides [3]. Most importantly, aromatherapy and massage are used in clinical settings [4,5] and in sports medicine [6] as complementary and alternative therapies. Aromatherapy massages are administered by diluting essential oils with carrier oils. For example, plant-derived oils (e.g. almond oil and jojoba oil) have been widely used as skin emollients and moisturizers [7,8,9,10,11].

Although numerous studies on massage therapy as a complementary or alternative therapy or in the context of sports medicine have been carried out, limited information is available regarding the benefits of massage oils or carrier oils for the skin. Furthermore, in several review articles, the type of massage oil used was not indicated [6,12,13,14,15,16,17]. Hence, especially regarding aromatherapy research, the pharmaceutically active ingredients contained in each essential oil are expected to exert effects, whereas the effect of the carrier oil, used to dilute the essential oil, is expected to be negligible. Similarly, studies on sports medicine primarily focus on the direct effect of the massage, even though the physiological effects of the massage oil itself are often not considered. The common carrier oils that are now used for aromatherapy massages are jojoba oil, grape seed oil, macadamia nut oil, and sweet almond oil. Among these, jojoba oil is one of the most widely used carrier oils worldwide.

Although jojoba oil is often categorized as an “oil” because of its pale yellow, transparent, liquid appearance, it is actually a liquid wax ester. Wax esters are generally straight-chain esters of mono-unsaturated long chain fatty acids and fatty alcohols. The wax esters produced by jojoba are very similar to sebum produced naturally by the human skin. Human sebum is comprised of approximately 33% triglycerides, 28% free fatty acids, 25% wax esters, 10% squalene, 2% cholesterol esters, and 4% cholesterol [18]. Further, wax esters exert moisturizing effects and impart softness to the skin.

Jojoba (*Simmondsia chinensis*) is the only plant species known for synthesizing liquid wax, which constitutes approximately 40–60% of the dry weight of mature jojoba seeds [19]. In general, plant-derived oils are rich in triglycerides; therefore, jojoba oil differs from other seed oils due to the fact that it contains primarily liquid wax, rather than triglycerides [20]. Although jojoba oils are widely used as carrier oils, limited information is available regarding their effect on blood biochemical parameters. This study aimed to investigate the effect of transdermal administration of jojoba oil on blood biochemical parameters in mice. Furthermore, we analyzed the expression of lipid metabolism-related genes in various tissues/organs after this treatment.

## 2. Materials and Methods

### 2.1. Ethics Statement

All animals were cared for in accordance with Law No. 105 and Notification No. 6 of the Japanese Government, and all animal experiments were carried out with the approval of the Animal Experimentation Committee of Tokai University (permission #171096 and #181020).

### 2.2. Animals

Seven-week-old male hairless mice (Hos-HR-1) were purchased from Hoshino Laboratory Animals, Inc. (Bando, Japan) through Japan SLC, Inc. (Hamamatsu, Japan), and housed at the Department of Laboratory Animal Science, the Support Center for Medical Research and Education, Tokai University, for at least 1 week. All mice were 8 weeks of age (26.9–32.3 g; average = 29.8 g) during the experiments. All mice were housed under specific pathogen-free conditions with a standard commercial diet (Clea, Tokyo, Japan) and water was provided *ad libitum* until just before euthanasia. The mice were housed at 22–24 °C with 50–60% relative humidity, under a light-dark cycle (lights on at 08:00 and off at 20:00).

### 2.3. Experimental Protocol

On the day of the experiment, all mice were randomly divided into two groups of six animals each, based on the different topical applications as follows: the naïve control group (26.9–32.3 g; average = 29.7 g) and the undiluted jojoba oil group (28.9–31.7 g; average = 29.9 g). Jojoba oil is a fatty acid-containing wax ester and is different from common vegetable oils, rich in triglycerides. Based on the manufacturer’s package insert, the ingredients of jojoba oil used in this study are listed in Table 1. Mice were topically administered 4 μL of jojoba oil (Kenso-Igakusha, Yamanashi, Japan; Lot. GL15A) per gram of body weight to the dorsal area, 30 min before euthanasia. The jojoba oil was evenly spread on the dorsal skin. After topical application, the mice were housed separately and monitored for adverse effects caused by oil application, in the form of skin rashes (erythema toxicum, dermatitis, etc.).

The sampling time was determined based on previous studies, wherein the time-dependent concentration of the active ingredient in blood was examined using hairless mice [21] and humans [22], after transdermal absorption of the essential oil. On preliminary analysis, an increase in sampling time affected lipid metabolism. Hence, all tissues were sampled from 9 a.m. to 11 a.m., so as not to affect lipid metabolism among all mice. Hairless mice were used because there was no risk of scratching the dorsal skin prior to transdermal administration, due to the process of hair removal.

### 2.4. Biochemical Analysis of Serum

Serum levels of albumin (ALB), blood urea nitrogen (BUN), creatine (CRE), uric acid (UA), aspartate aminotransaminase (AST), alanine aminotransaminase (ALT), alkaline phosphatase (ALP), creatine kinase (CK), total cholesterol (T-CHO), triglyceride (TG), phospholipid (PL), non-esterified fatty acids (NEFA), low-density lipoprotein cholesterol (LDL-C), high-density lipoprotein cholesterol (HDL-C), total bile acid (TBA), glucose (GLU), lactic acid (LA), and total ketone body (T-KB) were measured by Oriental Yeast Co., Ltd. (Tokyo, Japan).

### 2.5. RNA Isolation and Gene Expression Analysis Using Real-Time Polymerase Chain Reaction (Real-Time PCR)

Immediately after harvesting tissue/organ samples, each sample was frozen in liquid nitrogen and stored at −80 °C until RNA extraction, including the liver, white adipose tissue (WAT) of the epididymis, skin, brown adipose tissue (BAT), plantaris muscle, and the heart. Based on the manufacturers’ instructions, total RNA was extracted from the liver using the RNeasy Mini Kit (Qiagen, Valencia, CA, USA), total RNA from the heart, plantaris muscle, and skin were extracted using the RNeasy Fibrous Tissue Mini Kit (Qiagen), and total RNA from WAT and BAT were extracted using the RNeasy Lipid Tissue Mini Kit (Qiagen).

The purity and the quality of total RNA from each sample were assessed based on the optical density 260:280 nm ratio, using a NanoDrop spectrophotometer (Nano-Drop Technologies, Wilmington, DE, USA). Total RNA was reverse-transcribed to cDNA using the High Capacity cDNA Reverse Transcription Kit (Applied Biosystems, Foster, CA, USA), in accordance with the manufacturer’s instructions. PCR was carried out using the StepOnePlus system (Applied Biosystems, Foster City, CA, USA), using the Fast SYBR^®^ Green Master Mix (Applied Biosystems, Foster City, CA, USA). The cycling conditions were as follows: 10 s at 95 °C for denaturation, followed by 45 cycles at 95 °C for 5 s, 57 °C for 10 s (annealing), and, lastly, 72 °C for 10 s.

Data were normalized to β-actin levels as an internal standard, using the calibration curve method. Genes and primers are listed in Table 2.

### 2.6. Statistical Analysis

The results are expressed as mean ± standard error (SE). Statistical analyses were performed using the SPSS 24.0 statistical software package (SPSS Japan Inc., Tokyo, Japan). For inter-group comparisons of means and to confirm the normality in each group and each item, the Shapiro-Wilk test was performed. Unpaired *t*-tests were performed when both groups displayed a normal distribution and Mann-Whitney *U* tests were performed when either of the two groups did not display a normal distribution. The correlation between gene expression in various tissues/organs and serum NEFA levels was assessed based on the Spearman’s correlation coefficient (*r*). *P*-values less than 0.05 were considered statistically significant, whereas *P*-values less than 0.1 were considered to indicate a significant tendency. All statistical tests were two-tailed.

## 3. Results

### 3.1. Effects on Serum Biochemical Data 30 Minutes after Transdermal Administration of Jojoba Oil

To examine the association between serum biochemical data and the transdermal administration of jojoba oil, serum lipid concentrations, including those of T-CHO, TG, PL, and NEFA, also known as free fatty acids (FFA), were measured. As shown in Table 3, serum NEFA levels were significantly increased in comparison to those in the control group (*p* < 0.05). In contrast, no significant differences were observed between the two groups in serum T-CHO, TG, and PL. However, other biochemical parameters (ALB, BUN, CRE, etc.) remained unchanged. Together, these results indicate that 30 min after jojoba oil administration, serum lipid levels increased because of increased NEFA.

### 3.2. Changes in Expression Levels of Lipid Metabolism-Related Genes in Various Tissues/Organs after Transdermal Administration of Jojoba Oil

To investigate the cause of increased serum NEFA, we performed real-time PCR to evaluate expression levels of the following in various tissues/organs: (1) lipid degradation-related genes; (2) fatty acid trafficking-related genes; (3) lipogenesis-related genes. The percentage values for changes in gene expression in various tissues/organs relative to those in the control group are shown in Table 4.

#### 3.2.1. Changes in the Expression of Lipid Degradation-Related Genes

TGs are produced by the esterification of NEFA with glycerol, which is produced by sugar and alcohol metabolism. TGs are stored under the skin and around the viscera, and are metabolized into fatty acids, which serve as energy sources. Both ATGL and HSL enzymes are involved in TG degradation. *Atgl* was significantly upregulated in the liver 30 min after jojoba oil administration (*p* < 0.05), but there was no significant change seen in other tissues/organs. In contrast, there were no significant changes in *Hsl* expression in any tissues/organs 30 min after jojoba oil administration. Similarly, no changes were observed in the expression of *Lpl* which is involved in lipid uptake, and *Cpt-1α*, which regulates β-oxidation.

#### 3.2.2. Changes in the Expression of Fatty Acid Trafficking-Related Genes

Since fatty acids in the bloodstream are taken up by cells via CD36, FABPpm, and FATP family proteins, we examined the expression of these genes. *FATP-3* was significantly upregulated in the liver (*p* < 0.05) and *FATP-4* was significantly downregulated (*p* < 0.05). However, *FABPpm*, *FATP-1*, *FATP-3*, and *FATP-4* were downregulated in the skin of mice from the experimental group (*p* = 0.067, 0.074, 0.076, and 0.082, respectively).

#### 3.2.3. Changes in the Expression of Lipogenesis-Related Genes

To assess the expression levels of lipogenesis-related genes, the expression levels of *Fas*, *Acc-1*, *Acc-2*, and *Scd-1* were evaluated via real-time PCR analysis. *Fas* and *Acc-1* in the BAT were significantly downregulated in the experimental group (*p* < 0.05). Additionally, *Acc-2* in the skin and the plantaris muscle was significantly downregulated in the experimental group (*p* < 0.05). In association with lipogenesis, *Srebp-1c*, coding a transcription factor associated with fatty acid synthesis by upregulating *Scd-1*, was significantly downregulated in the skin, BAT, and plantaris muscle (*p* < 0.05). However, *Scd-1* was not upregulated in any of these tissues/organs.

### 3.3. Correlation between Lipid Metabolism-Related Genes Expressed in Various Tissues/Organs and Serum NEFA Levels after Transdermal Administration of Jojoba Oil

To confirm that the alterations in gene expression levels in the associated tissues/organs were correlated with elevated serum NEFA levels, we examined the relationship between gene expression levels in various tissues/organs and serum NEFA levels (Table 5). The results of the analysis are described as follows for correlations among lipid degradation-related genes, fatty acid trafficking-related genes, lipogenesis-related genes, and serum NEFA levels.

#### 3.3.1. Correlations between Lipid Degradation-Related Genes Expressed in Various Tissues/Organs and Serum NEFA Levels after Transdermal Administration of Jojoba Oil

**Figure 1:**
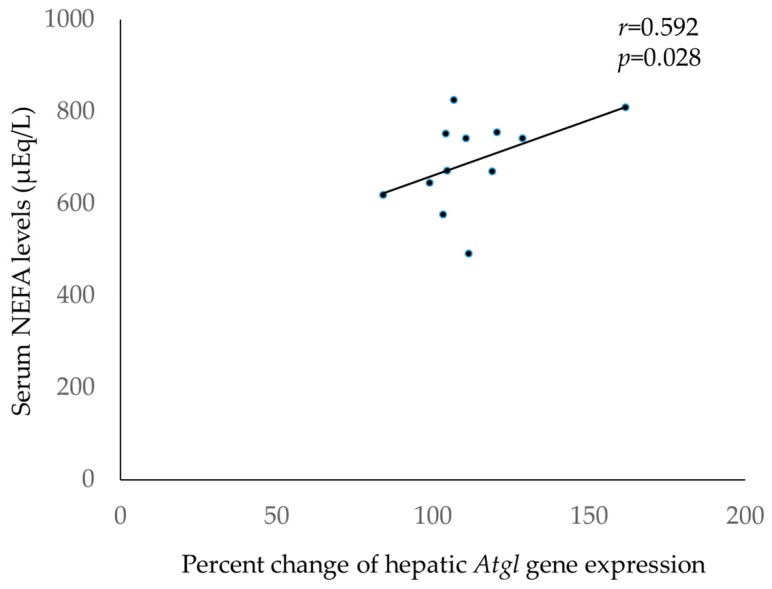
Correlation between serum non-esterified fatty acid (NEFA) levels and liver *Atgl* expression levels 30 min after topical application of jojoba oil. Serum NEFA levels were significantly correlated with liver *Atgl* expression levels (*r* = 0.592, *p* = 0.028).

As shown in Table 5 and Figure 1, liver *Atgl* expression levels were positively and significantly correlated with serum NEFA levels (*r* = 0.592, *p* < 0.05), and skin *Hsl* expression levels tended to be negatively correlated with serum NEFA levels (*r* = −0.427, *p* = 0.083).

#### 3.3.2. Correlations between Fatty Acid Trafficking-Related Genes Expressed in Various Tissues/Organs and Serum NEFA Levels after Transdermal Administration of Jojoba Oil

Liver *Cd36* expression levels tended to be positively correlated with serum NEFA levels (*r* = 0.528, *p* = 0.095), whereas skin *FATP-3* expression levels tended to be negatively correlated with serum NEFA levels (*r* = −0.501, *p* = 0.097).

#### 3.3.3. Correlations between Lipogenesis-Related Genes Expressed in Various Tissues/Organs and Serum NEFA Levels after Transdermal Administration of Jojoba Oil

Cardiac *Scd-1* expression levels were positively and significantly correlated with serum NEFA levels (*r* = 0.581, *p* < 0.05).

#### 3.3.4. Correlations between Nuclear Transcription Factors Expressed in Various Tissues/Organs and Serum NEFA Levels after Transdermal Administration of Jojoba Oil

Cardiac *Lpin-1* expression levels were negatively and significantly correlated with serum NEFA levels (*r* = −0.609, *p* < 0.05). Furthermore, liver *Sirt-1* and *Lpin-1* expression levels tended to be positively correlated with serum NEFA levels (*r* = 0.592, *p* = 0.055; *r* = 0.583, *p* = 0.060, respectively), whereas *Srebp-1c* expression levels in the plantaris muscle tended to be negatively correlated with serum NEFA levels (*r* = −0.567, *p* = 0.054).

## 4. Discussion

The results from the present study show that serum NEFA levels are elevated upon topical administration of jojoba oil in mice. Jojoba oil has anti-inflammatory effects, anti-skin-aging effects, wound healing effects, antioxidant effects, antibacterial effects, and antifungal effects [7,23,24,25]. However, no studies have reported the effects of transdermal application of jojoba oil on lipid metabolism. To our knowledge, this is the first report on the analysis of serum biochemical data after transdermal administration of jojoba oil and gene expression analysis of lipid metabolism-related genes in various tissues/organs. In the present study, serum NEFA levels were significantly increased 30 min after topical application of jojoba oil (Table 3).

The potential mechanisms underlying the elevation in serum NEFA levels are described as follows. As previously indicated, jojoba oil is composed of a liquid wax ester, which is an ester comprised of fatty acids (73.4% eicosenoic acid, 14.7% erucic acid, and 8.3% oleic acid) and fatty alcohols. Thus, we presume that these fatty acids are absorbed into the skin and, subsequently, modify the expression of transcription factors and genes correlated with fatty acid metabolism, through a mechanism of substrate-dependent gene expression.

Since NEFA levels were increased in the jojoba oil-treated group based on an examination of serum biochemistry, the relative expression of lipid metabolism-related genes was analyzed. NEFA is rapidly metabolized in the blood and has a half-life for 1–2 min. The following events might elevate serum NEFA levels: (1) TG degradation in the WAT, and decomposition of intrahepatic TG droplets into fatty acids and glycerol; (2) decreased intracellular uptake of fatty acids into cells by CD36, FABPpm, and the FATP family; (3) a fatty acid supply to blood via lipogenesis. Therefore, we performed real-time PCR to evaluate the expression levels in various tissues/organs of (1) lipid degradation-related genes, (2) fatty acid trafficking-related genes, and (3) lipogenesis-related genes.

Since TG degradation involves both ATGL and HSL enzymes, we analyzed the gene expression levels of both *Atgl* and *Hsl*. Regarding lipolysis-related gene expression, *Atgl* was upregulated only in the liver, and no change was observed in the WAT, skin, BAT, plantaris muscle, and heart (Table 4). Furthermore, a significant positive correlation was observed between the relative expression levels of *Atgl* in the liver, and serum NEFA levels after topical application of jojoba oil (Figure 1). Therefore, degradation of lipid droplets in the liver may contribute to the increase in serum NEFA levels after this treatment. Because ATGL is a triacylglycerol hydrolase that promotes the lipolysis of stored fat [26], an association between the upregulation of *Atgl* in the liver and elevated serum NEFA levels indicates that hepatic lipolysis caused the release of NEFA into the blood, and increased serum NEFA levels.

Since fatty acids in the bloodstream are taken up by cells via CD36, FABPpm, and the FATP family proteins, the expression of these genes was examined. As a result, after 30 min of topical application of jojoba oil, *FABPpm*, *FATP-1*, *FATP-3*, and *FATP-4* tended to be downregulated in the skin. The reason for this might be related to SREBP-1, which regulates the expression levels of *Lpin-1*. Lipin 1 is a co-factor required for PPAR-α activation [27], and PPAR-α regulates *FATP* mRNA expression [28]. Therefore, the significant decrease in expression levels of *Srebp-1c*, *Lpin-1*, and *Ppar-a* in the skin could be related to the fact that the expression levels of fatty acid trafficking-related genes also tended to be downregulated in the skin. Considering that the skin is the largest organ, the decreased expression of four fatty acid trafficking-related genes in the skin might have potentially increased serum NEFA levels. In this study, skin *FATP-2*, *FATP-5*, and *FATP-6* expression levels were considered at the lower limit of determination. These results are concurrent with those of previous studies [29]. In the liver, although *FATP-3* was significantly upregulated, *FATP-4* was significantly downregulated. However, Schaffer reported that *FATP* mRNA is highly expressed in skeletal muscle, the heart, and body fat, but found at low levels in the livers of normal mice [30]. Therefore, hepatic lipid intake might not be affected, when taking into account the offset of increased hepatic *FATP-3* expression levels by decreased hepatic *FATP-4* expression levels.

Regarding lipogenesis-related gene expression, acetyl-CoA carboxylase (ACC-1, ACC-2) promotes fatty acid synthesis. In addition, fatty acid synthase (FAS) is an important rate-limiting step in lipogenesis. ACC catalyzes the conversion of acetyl CoA to malonyl-CoA, which is a potent inhibitor of carnitine palmitoyl transferase-1 (CPT-1). Therefore, ACC-2 indirectly prevents the influx of fatty acids into the mitochondria and subsequent β-oxidation [31]. Thus, it is possible that β-oxidation of mitochondrial fatty acid increased after 30 min, subsequent to jojoba oil administration in the skin and plantaris muscle, where *Acc-2* gene expression was significantly reduced. Since SREBP-1c is a master regulator of lipogenesis [32,33], the reason for the significantly downregulated expression levels of lipogenesis-related genes in the skin, BAT, and plantaris muscle could be related to downregulated expression of *Srebp-1c*.

To date, several studies have examined the effects of vegetable oils, in the presence or absence of massage, on neonates, and reported that neonatal growth is promoted through oil massages [34,35,36,37]. Although the mechanisms of action are yet unclear, triglycerides and fatty acids are suggested to be significantly elevated upon topical application of vegetable oils, which may potentially serve as a nutrient source and, hence, promote neonatal growth. Moreover, some studies have suggested that topical application of vegetable oils is significantly associated with changes in vagal tone during massaging, and changes in gastric motility after a massage [38]. Unlike TG-based vegetable oils, jojoba oil used herein is based on wax esters. However, the significant increase in fatty acids after topical application is a feature common to both jojoba and vegetable oils.

The significant increase in serum fatty acid levels after topical application of jojoba oil provides novel insights into the potential applications of this oil to help promote the growth of neonates, improve nutrition among elderly individuals, improve endurance exercises among athletes, and improve the therapeutic effects of physical therapy. Further studies are needed to clarify whether the serum fatty acids, which increased upon topical application of jojoba oil, are used as energy substrates.

This study has potential limitations. First, the dose of oil administered to the mice was larger than the dose usually administered to humans for massage or rubbing (equivalent to 240 mL of oil for a 60-kg person). However, massage and rubbing could enhance lipid absorption. Since massage or rubbing increases blood flow and skin temperature, it has been reported that these techniques are likely to alter the stratum corneum structure and enhance transdermal absorption [39]. Second, the small sample number could hinder the detection of some slight differences. Furthermore, the skin has numerous functions. However, the most obvious function is both a physical and a biological defense. Therefore, not all compounds can penetrate the skin, and a characteristic molecular weight of 500 Da or less is important for transdermal absorption [40]. As shown in Table 1, the molecular weights of fatty acids contained in jojoba oil are less than 500 Da. However, fatty acids contained in jojoba oil are present as wax esters, and it remains unclear whether wax esters are degraded into fatty acids and alcohols by the skin microflora. Moreover, according to confocal microscopic studies examining the penetration of jojoba oil into human skin, jojoba oil penetrates only into the outermost layers of the stratum corneum [10,11,41]. Therefore, it is an open question whether the fatty acid constituting the wax ester, per se, are percutaneously absorbed into the blood. Although certain aspects remain unknown, it is clear that specific constituents of jojoba oil penetrated the skin after topical application.

## 5. Conclusions

This study shows that serum NEFA levels are elevated 30 min after transdermal administration of jojoba oil, thus indicating that certain constituents of jojoba oil penetrate the skin. The mechanism underlying elevated serum NEFA levels might be comprised of both enhanced lipolysis via *Atgl* upregulation in the liver, and reduced fatty acid trafficking via *FABPpm*, *FATP1*, *FATP3*, and *FATP4* downregulation in the skin. Further studies are needed to clarify what constituents of jojoba oil can be absorbed transdermally.

## Figures and Tables

**Table 1:** Fatty acid composition of jojoba oil.

Name	MW [g/mol]	Content [%]
Eicosenoic acid	310.51	73.4
Erucic acid	338.57	14.7
Oleic acid	282.47	8.3

MW: molecular weight.

**Table 2:** Primers used for real-time polymerase chain reaction analysis.

Name	Accession no.	Forward	Reverse
*Atgl*	NM_025802.3	TGTGGCCTCATTCCTCCTAC	TCGTGGATGTTGGTGGAGCT
*Hsl*	NM_010719.5	GCTGGGCTGTCAAGCACTGT	GTAACTGGGTAGGCTGCCAT
*Lpl*	NM_008509.2	CCAATGGAGGCACTTTCCA	TGGTCCACGTCTCCGAGTC
*Cpt-1a*	XM_006531658.3	CCAGGCTACAGTGGGACATT	AAGGAATGCAGGTCCACATC
*Cd36*	NM_001159558.1	CCGGGCCAACGTAGAAAACA	CCTCCAAACACAGCCAGGAC
*FABPpm*	NM_010325.2	AGCGGCTGACCAAGGAGTT	GACCCCTGCCACGGAGAT
*FATP-1*	NM_011977.4	GGCTCCTGGAGCAGGAACA	ACGGAAGTCCCAGAAACCAA
*FATP-2*	NM_011978.2	TTCGGGAACCACAGGTCTTC	GCAAGGCTTGTCCCATACCTT
*FATP-3*	NM_001316688.1	CAGCTCTACAGCCATGTTTCTGA	CAAAGATTCCTGGAGCCTGAGA
*FATP-4*	NM_011989.5	GGCTTCCCTGGTGTACTATGGAT	ACGATGTTTCCTGCTGAGTGGTA
*FATP-5*	NM_009512.2	TTTCTGGGGTTGGCCAAGTT	TGGCCAAGGTAGAAGCAGTG
*FATP-6*	NM_001081072.1	GGCTTGAGGATGCCGCTTA	GTACTCTGGGCTCATGCTATGAAGT
*Acc-1*	XM_011248667.1	ATTGGGCACCCCAGAGCTA	CCCGCTCCTTCAACTTGCT
*Acc-2*	XM_006530113.3	GGGCTCCCTGGATGACAAC	TTCCGGGAGGAGTTCTGGA
*Fas*	NM_007988.3	CCTGGATAGCATTCCGAACCT	AGCACATCTCGAAGGCTACACA
*Scd-1*	NM_009127.4	TTCTTGCGATACACTCTGGTGC	CGGGATTGAATGTTCTTGTCGT
*Srebp-1c*	XM_006532716.2	GGAGCCATGGATTGCACATT	GGCCCGGGAAGTCACTGT
*Lpin-1*	NM_001355598.1	CCATTCACAGCGAGTCTTCA	TGGAAGGGGAATCTGTCTTG
*Ppar-a*	XM_006520624.3	TCTGTGGGCTCACTGTTCT	AGGGCTCATCCTGTCTTTG
*Sirt-1*	NM_001159289.2	GCAACAGCATCTTGCCTGAT	GTGCTACTGGTCTCACTT
*Actb*	NM_007393.5	CCTCCCTGGAGAAGAGCTATG	TTACGGATGTCAACGTCACAC

*Atgl*, adipose triglyceride lipase. *Hsl*, hormone sensitive lipase. *Lpl*, lipoprotein lipase. *Cpt-1a*, carnitine palmitoyl transferase-1α. *Cd36*, cluster of differentiation 36. *FABPpm*, plasma membrane fatty acid binding protein. *FATP-1*, fatty acid transport protein-1. *FATP-2*, fatty acid transport protein-2. *FATP-3*, fatty acid transport protein-3. *FATP-4*, fatty acid transport protein-4. *FATP-5*, fatty acid transport protein-5. *FATP-6*, fatty acid transport protein-6. *Fas*, fatty acid synthase. *Acc-1*, acetyl coenzyme A carbocylase-1. *Acc-2*, acetyl coenzyme A carbocylase-2. *Scd-1*, stearoyl-CoA desaturase-1. *Ppar-a*, peroxisome proliferator-activated receptor-α. *Srebp-1c*, sterol regulatory element binding protein-1c. *Sirt-1*, sirtuin-1. *Lpin-1*, lipin-1. *Actb,* β-actin.

**Table 3:** Comparison of serum biochemical parameters between the naïve control and jojoba oil topical application groups.

		Control	Jojoba Oil
ALB	(g/dL)	2.8 ± 0.1	2.9 ± 0.0
BUN	(mg/dL)	25.9 ± 1.1	23.8 ± 1.0
CRE	(mg/dL)	0.1 ± 0.0	0.1 ± 0.0
UA	(mg/dL)	2.0 ± 0.2	2.2 ± 0.1
AST	(IU/L)	99.5 ± 15.4	87.0 ± 6.8
ALP	(IU/L)	401.7 ± 21.9	414.7 ± 17.0
CK	(IU/L)	388.0 ± 102.0	216.7 ± 45.1
T-CHO	(mg/dL)	73.8 ± 2.3	70.0 ± 1.9
TG	(mg/dL)	67.5 ± 7.7	62.0 ± 6.7
PL	(mg/dL)	154.3 ± 4.0	148.7 ± 5.5
NEFA	(μEq/L)	626.2 ± 36.0	757.7 ± 22.8 *
LDL-C	(mg/dL)	4.8 ± 0.4	4.3 ± 0.6
HDL-C	(mg/dL)	47.0 ± 1.5	44.3 ± 1.4
TBA	(μmol/L)	1.2 ± 0.2	1.0 ± 0.0
GLU	(mg/dL)	239.5 ± 17.5	223.7 ± 12.2
LA	(mg/dL)	81.0 ± 6.9	66.8 ± 5.3
T-KB	(μmol/L)	317.0 ± 25.5	338.7 ± 25.4

ALB, albumin. BUN, blood urea nitrogen. CRE, creatine. UA, uric acid. AST, asparatate aminotransaminase. ALP, alkaline phosphatase. CK, creatine kinase. T-CHO, total cholesterol. TG, triglyceride. PL, phospholipid. NEFA, non-esterified fatty acids. LDL-C, low-density lipoprotein cholesterol. HDL-C, high-density lipoprotein cholesterol. TBA, total bile acid. GLU, glucose. LA, lactic acid. T-KB, total ketone body. Values are means ± SE, *n* = 6. * *p* < 0.05.

**Table 4:** Percentage values of changes in gene expression in various tissues/organs.

	Liver	WAT	Skin	BAT	Plantaris Muscle	Heart
Lipid degradation						
*Atgl*	123.2 ± 8.0 *	138.4 ± 22.8	68.0 ± 3.6	102.5 ± 3.6	74.5 ± 8.2	92.4 ± 3.1
*Hsl*	95.0 ± 4.0	134.7 ± 21.3	55.4 ± 4.9 ^†^	114.8 ± 2.9	84.3 ± 5.2	80.4 ± 9.1
*Lpl*	94.9 ± 3.8	135.7 ± 20.8	90.7 ± 15.4	99.0 ± 8.2	81.2 ± 6.1	90.0 ± 5.4
*Cpt-1a*	112.1 ± 6.8	112.9 ± 36.0	103.7 ± 8.2	89.6 ± 3.5	80.9 ± 10.6	99.3 ± 6.6
Fatty acid trafficking						
*Cd36*	140.4 ± 23.6	113.1 ± 16.4	108.6 ± 10.0	110.9 ± 3.5	94.6 ± 9.8	102.1 ± 6.4
*FABPpm*	101.6 ± 9.4	47.6 ± 34.4	64.7 ± 7.4 ^†^	128.3 ± 34.2	88.5 ± 6.4	94.6 ± 6.2
*FATP-1*	83.0 ± 4.9	89.1 ± 16.3	30.0 ± 3.8 ^†^	103.6 ± 6.6	61.0 ± 22.1	96.6 ± 3.4
*FATP-2*	112.6 ± 13.8	77.2 ± 52.1	N.D.	123.8 ± 11.6	87.2 ± 79.1	36.7 ± 20.9
*FATP-3*	198.4 ± 21.5 **	100.8 ± 15.9	43.6 ± 4.8 ^†^	84.2 ± 4.1	76.5 ± 7.0	140.7 ± 30.1
*FATP-4*	80.8 ± 3.9 *	84.2 ± 26.3	69.0 ± 5.0 ^†^	97.4 ± 4.9	76.3 ± 8.7	107.5 ± 8.4
*FATP-5*	112.6 ± 11.0	53.3 ± 29.1	N.D.	39.7 ± 6.9	N.D.	106.6 ± 36.7
*FATP-6*	N.D.	35.2 ± 15.5	N.D.	16.3 ± 4.8	65.5 ± 20.1	40.9 ± 14.5
Lipogenesis						
*Acc-1*	91.4 ± 6.6	119.7 ± 19.0	53.3 ± 3.6 ^†^	87.6 ± 3.7 *	103.2 ± 5.8	58.7 ± 15.0
*Acc-2*	81.5 ± 6.6	138.0 ± 22.6	40.4 ± 3.4 *	100.2 ± 5.2	74.4 ± 5.4 ^*^	91.5 ± 5.6
*Fas*	89.7 ± 9.9	99.3 ± 16.9	85.3 ± 4.0	87.5 ± 2.6 *	97.2 ± 12.5	51.1 ± 20.7
*Scd-1*	81.0 ± 7.1	133.4 ± 28.0	81.9 ± 16.9	96.0 ± 3.8	158.6 ± 28.5 ^†^	81.7 ± 29.3
Nuclear transcrption factors						
*Srebp-1c*	94.6 ± 7.7	95.5 ± 5.5	52.6 ± 9.4 **	87.2 ± 2.1 **	58.1 ± 2.6 *	78.0 ± 7.0
*Lpin-1*	284.8 ± 68.6 *	130.5 ± 14.7	43.1 ± 5.0 *	95.5 ± 3.2	75.9 ± 7.6	76.4 ± 4.2
*Ppar-a*	92.1 ± 8.3	125.6 ± 16.5	14.3 ± 4.2 *	129.8 ± 12.2	119.1 ± 44.0	85.9 ± 6.3
*Sirt-1*	157.6 ± 16.7 *	90.7 ± 9.7	127.1 ± 6.1 ^†^	89.4 ± 2.6	116.2 ± 13.2	112.8 ± 7.2

*Atgl*, adipose triglyceride lipase. *Hsl*, hormone sensitive lipase. *Lpl*, lipoprotein lipase. *Cpt-1a*, carnitine palmitoyl transferase-1α. *Cd36*, cluster of differentiation 36. *FABPpm*, plasma membrane fatty acid binding protein. *FATP-1*, fatty acid transport protein-1. *FATP-2*, fatty acid transport protein-2. *FATP-3*, fatty acid transport protein-3. *FATP-4*, fatty acid transport protein-4. *FATP-5*, fatty acid transport protein-5. *FATP-6*, fatty acid transport protein-6. *Fas*, fatty acid synthase. *Acc-1*, acetyl coenzyme A carbocylase-1. *Acc-2*, acetyl coenzyme A carbocylase-2. *Scd-1*, stearoyl-CoA desaturase-1. *Ppar-a*, peroxisome proliferator-activated receptor-α. *Srebp-1c*, sterol regulatory element binding protein-1c. *Sirt-1*, sirtuin-1. *Lpin-1*, lipin-1. WAT, white adipose tissue. BAT, brown adipose tissue. N.D., not detected. Values are means ± SE, n = 6. ^†^ *p* < 0.1, * *p* < 0.05, ** *p* < 0.01.

**Table 5:** Associations among gene expression levels in various tissues/organs and serum non-esterified fatty acid (NEFA) levels assessed based on correlation coefficient.

	Liver	WAT	Skin	BAT	Plantaris Muscle	Heart
Lipid degradation						
*Atgl*	0.592 *	0.392	−0.385	0.095	−0.294	−0.179
*Hsl*	−0.178	0.025	−0.427 ^†^	0.235	−0.179	0.196
*Lpl*	−0.046	0.231	0.123	0.277	−0.018	−0.469
*Cpt-1a*	0.133	−0.063	0.168	−0.196	−0.098	−0.200
Fatty acid trafficking						
*Cd36*	0.528 ^†^	−0.035	−0.032	0.179	−0.287	−0.263
*FABPpm*	0.049	−0.081	−0.133	0.182	−0.266	−0.375
*FATP-1*	0.305	0.028	−0.284	0.238	−0.203	−0.298
*FATP-2*	0.098	0.091	N.D.	0.091	−0.071	0.395
*FATP-3*	0.452	0.312	−0.501 ^†^	−0.361	−0.168	0.462
*FATP-4*	−0.126	−0.319	−0.357	−0.004	−0.413	−0.231
*FATP-5*	0.203	−0.238	N.D.	−0.056	N.D.	0.035
*FATP-6*	N.D.	−0.207	N.D.	−0.132	−0.237	−0.074
Lipogenesis						
*Acc-1*	0.025	0.277	−0.147	−0.137	0.025	0.347
*Acc-2*	0.770	0.326	−0.280	0.109	−0.333	−0.392
*Fas*	−0.084	−0.004	0.070	−0.228	0.294	0.487
*Scd-1*	0.105	0.259	−0.123	0.155	0.490	0.581 *
Nuclear transcrption factors						
*Srebp-1c*	−0.389	−0.119	−0.438	−0.497	−0.567 ^†^	−0.291
*Lpin-1*	0.583 ^†^	0.228	−0.543	0.130	−0.158	−0.609 *
*Ppar-a*	−0.123	0.028	−0.452	0.109	0.053	−0.424
*Sirt-1*	0.592 ^†^	0.238	0.308	−0.333	−0.238	0.147
